# Patterns of *Plasmodium homocircumflexum* virulence in experimentally infected passerine birds

**DOI:** 10.1186/s12936-019-2810-2

**Published:** 2019-05-21

**Authors:** Mikas Ilgūnas, Dovilė Bukauskaitė, Vaidas Palinauskas, Tatjana Iezhova, Karin Fragner, Elena Platonova, Herbert Weissenböck, Gediminas Valkiūnas

**Affiliations:** 1 Nature Research Centre, Akademijos 2, 08412 Vilnius, Lithuania; 2 Institute of Pathology, University of Veterinary Medicine Vienna, 1210 Vienna, Austria

**Keywords:** Avian malaria, *Plasmodium*, Birds, Phanerozoites, Pathology

## Abstract

**Background:**

Avian malaria parasites (genus *Plasmodium*) are cosmopolitan and some species cause severe pathologies or even mortality in birds, yet their virulence remains fragmentally investigated. Understanding mechanisms and patterns of virulence during avian *Plasmodium* infections is crucial as these pathogens can severely affect bird populations in the wild and cause mortality in captive individuals. The goal of this study was to investigate the pathologies caused by the recently discovered malaria parasite *Plasmodium homocircumflexum* (lineage pCOLL4) in four species of European passeriform birds.

**Methods:**

One cryopreserved *P. homocircumflexum* strain was multiplied and used for experimental infections. House sparrows (*Passer domesticus*), common chaffinches (*Fringilla coelebs*), common crossbills (*Loxia curvirostra*) and common starlings (*Sturnus vulgaris*) were exposed by subinoculation of infected blood. Experimental and control groups (8 individuals in each) were observed for over 1 month. Parasitaemia, haematocrit value and body mass were monitored. At the end of the experiment, samples of internal organs were collected and examined using histological and chromogenic in situ hybridization methods.

**Results:**

All exposed birds were susceptible, with similar average prepatent period and maximum parasitaemia, yet virulence was different in different bird species. Mortality due to malaria was reported in chaffinches, house sparrows and crossbills (7, 5 and 3 individuals died respectively), but not in starlings. Exoerythrocytic meronts (phanerozoites) were observed in the brain of all dead experimental birds. Blockage of blood vessels in the brain led to cerebral ischaemia, invariably causing brain damage, which is likely the main reason of mortality. Phanerozoites were observed in parenchymal organs, heart and muscles of all infected individuals, except starlings.

**Conclusion:**

This study shows that *P. homocircumflexum* is generalist and the same lineage caused similar parasitaemia-related pathologies in different host species. Additionally, the mode of exo-erythrocytic development is different in different birds, resulting in different mortality rates. This should be taken into consideration in studies addressing pathology during avian malaria infections.

## Background

With the exception of Antarctica, agents of avian malaria (Plasmodiidae, Haemosporida) have been reported all over the world [1, 2]. In all, 55 species of these pathogens have been recognized [3], and many new agents of avian malaria were discovered recently [4–9]. Species of *Plasmodium* have complex life cycles, which remain incompletely investigated for the majority of these pathogens [10]. These parasites can cause severe health disorders in domestic, wild and captive birds, sometimes even leading to lethal malaria [10–14].

There are two main causes of pathology during avian malaria infections: blood pathology [12, 15] and organ damage due to phanerozoites—tissue meronts developing during secondary exo-erythrocytic merogony [11, 12, 16, 17]. During avian malaria, merozoites from erythrocytic meronts can induce exo-erythrocytic merogony (development of phanerozoites), and that is not the case during malaria in mammals. If blood pathology caused by erythrocytic stages of avian malaria parasites has been relatively well studied [1, 12], the damage caused by tissue stages and patterns of their occurrence remain insufficiently understood. Because phanerozoites develop in various non-specialized reticuloendothelial cells (macrophages, endothelial cells of capillaries), they can occur and cause damage of various organs in susceptible vertebrate hosts [10–12, 18, 19]. Due to the secondary exo-erythrocytic merogony, avian malaria may be a more virulent disease than human malaria. Additionally, the cause of virulence in avian malaria is more difficult to predict than during malaria in mammals due to unclear patterns of phanerozoite occurrence.

Recently, the issue of virulence of avian malaria pathogens has attracted much attention. However, majority of the investigations focused mainly on parasitaemia during *Plasmodium* infections [20–25]. These studies provided valuable information about blood-related pathological changes but are limited to truly evaluate mechanisms of the virulence during avian malaria due to the lack of information about pathology caused in organs. Knowledge about patterns of exo-erythrocytic development of malaria and other haemosporidian parasites remains insufficient. Most studies dealing with exo-erythrocytic development were carried out between 1930s and 1960s [10]—before molecular diagnostic techniques were introduced in avian malaria research. Application of molecular diagnostic methods showed that the diversity of parasites (both inter- and intra-species) is far greater than previously believed. Thus, it becomes even more difficult to address exo-erythrocytic development of particular parasites and their lineages, calling for the application of modern diagnostic tools and experimental research in avian malaria studies.

Phanerozoite stage is difficult to access in wild-caught naturally infected avian hosts, in which the longevity of malaria infection usually is unknown. Additionally, the exo-erythrocytic meronts also are sometimes difficult to visualize using the traditional histological approaches, particularly during light malaria infections. Molecular diagnostic tools have been developed and might aid with the detection and identification of phanerozoites [26, 27]. Several recent studies reported detection of exo-erythrocytic stages of avian malaria parasites, but these were mainly case reports [19, 28–30]. Patterns of the exo-erythrocytic development of avian *Plasmodium* species are still insufficiently understood. Experimental work would be helpful to develop this knowledge, which is crucial to answer questions related to bird health, treatment, ecology and possible threats to biodiversity.

Malaria parasite *Plasmodium homocircumflexum* (lineage pCOLL4) was recently discovered and described in Europe [8, 31]. A pilot study was conducted with the aim to investigate the effects of this infection on three individual birds belonging to three species [17]. The obtained data suggested that Eurasian siskins (*Carduelis spinus*), common crossbills (*Loxia curvirostra*) and common starlings (*Sturnus vulgaris*) were readily susceptible to *P. homocircumflexum* infections. Moreover, this parasite developed phanerozoites in these birds and was lethal in all tested bird individuals. This called for more a detailed investigation of pathology caused by *P. homocircumflexum* in avian hosts.

The goal of this study was to experimentally investigate (1) the dynamics of parasitaemia and the parasitaemia related health parameters (haematocrit value and body mass) during *P. homocircumflexum* (lineage pCOLL4) infection in four common European bird species (common crossbill, common starling, house sparrow *Passer domesticus* and common chaffinch *Fringilla coelebs*) and (2) the development of secondary exo-erythrocytic meronts and pathologies caused in these birds. Parasitaemia, haematocrit level and bird body mass were monitored at consistent time intervals, and birds were screened using histological and chromogenic *in* *situ* hybridization methods for detection of the exo-erythrocytic stages.

## Methods

### Study site

This study was carried out at the Biological Station of the Zoological Institute of the Russian Academy of Sciences on the Curonian Spit in the Baltic Sea (55°05′ N, 20°44′ E) during the months of May–August, 2015 and 2016. Juvenile wild birds (< 7 months old) were used. They were caught using funnel traps and mists nets and identified to species and age according to [32]. Prior to the experiments, all birds were screened for haemosporidian parasites using microscopic examination of blood films, and only non-infected birds were selected. The non-infected status of all birds prior the experiments was confirmed by polymerase chain reaction (PCR)-based testing in the laboratory as described below. Recipient birds were infected by subinoculation of *P. homocircumflexum* (lineage pCOLL4) infected blood and maintained until their death or the end of the experiment (between 40 and 64 days post exposure (DPE) in different experiments), at which point they were euthanized. The study aimed to maintain experiments until mortality was recognized in experimental groups, and that happened at different intervals in different bird species (see the “Results” section).

### Experimental design

All experimental bird species were unavailable in 1 year thus experiments were performed in two successive years using the same clone of malaria parasite. In 2015, juvenile house sparrows (*Passer domesticus*), common chaffinches (*Fringilla coelebs*) were available for experimental research. These bird species are abundant in Europe and were relatively easy to obtain in necessary numbers that year. Juvenile common crossbills (*Loxia curvirostra*) and common starlings (*Sturnus vulgaris*) were available for this experiment in 2016; these species were selected based on a pilot study [17], which showed that *P. homocircumflexum* develops phanerozoites in these birds. The common crossbills, common chaffinches and house sparrows were kept indoors in a vector-free room. The common starlings were kept outside in cages covered with a fine-mesh bolting silk preventing access of blood-sucking insects to birds. All birds were maintained at a natural light–dark photoperiod.

A strain of *P. homocircumflexum* (lineage pCOLL4, GenBank accession no. KC884250), originally isolated from a naturally infected red-backed shrike was used to infect the experimental birds. This strain was obtained from the biobank of the P. B. Šivickis Laboratory of Parasitology, Nature Research Centre, Vilnius, Lithuania [8]. Because of (1) the small size of donor birds and the resulting limited amount of blood, which could be withdrawal without damaging the bird and (2) the different number of mature meronts in infected blood of different donor birds during exposure, all experimental bird groups were exposed to different doses of infections (Table 1). That is why quantitative data of experiments with different bird species (level of parasitaemia, haematocrit value, body mass) should be used with caution for comparison between bird species. These data reflect parasite development within certain bird species after infection with certain dose of infection.

**Table 1:** Susceptibility of passerine birds to *Plasmodium homocircumflexum* (lineage pCOLL4) infection after experimental exposure

Bird species, group and infection dose	No. exposed (no. infected)	No. died	Prepatent period (days)	Maximum parasitemia^a^	Minimum hematocrit value^a^	Maximum weight, g^a^	No. of individuals with phanerozoites located in						
							Brain	Heart	Liver	Lung	Spleen	Kidney	Muscle
*Loxia curvirostra*													
Experimental (3 × 10^6^)^b^	8 (8)	7	4–8	3.3–23.3 (10)	12.5–33.3 (23.8)	37.4–45.4 (42.5)	7	8	8	8	8	8	8
Control	8 (0)	0	–	–	27.6–50 (43.7)	39.2–48.8 (43.9)	0	0	0	0	0	0	0
*Sturnus vulgaris*													
Experimental (9.5 × 10^5^)^b^	8 (8)	3	4–12	0.1–30 (13)	18.6–33.3 (28.15)	65.5–91.1 (80.2)	0	0	0	0	0	0	0
Control	8 (0)	1	–	–	31.4–37 (34.2)	71.8–83.8 (77.4)	0	0	0	0	0	0	0
*Passer domesticus*													
Experimental (8 × 10^5^)^b^	8 (8)	5	5	0.1–41.4 (9.4)	11.4–40 (28.1)	24.5–30.2 (28.4)	4	4	8	8	7	7	4
Control	8 (0)	2	–	–	28.6–45.5 (37.5)	26.8–31 (28.75)	0	0	0	0	0	0	0
*Fringilla coelebs*													
Experimental (5 × 10^5^)^b^	8 (8)	3	5	0.1–40.4 (11.5)	21–42 (31)	20.7–23.7 (22.)	2	2	4	7	4	4	2
Control	8 (0)	1	–	–	31–47.8 (40.3)	20.6–24.9 (23.1)	0	0	0	0	0	0	0

^a^Variation of individual parameters, followed in parentheses by the average for entire group

^b^Dose (total number of mature meronts) inoculated in each individual bird. It was difficult to standardize dose of infection in all experimental groups, mainly due to difficulty to standardize and calculate certain number of mature erythrocytic meronts in each inoculum, particularly in different year experiments. That is why comparison of quantitative data between bird species should be done with caution

In 2015 and 2016, one sample of this isolate was thawed according to [11] and used to multiply the parasite by passage in live birds. In 2015, 5 Eurasian siskins (*Carduelis spinus*) were exposed for multiplication of the same strain. First, the infected blood was obtained from 2 siskins, mixed and used to infect 8 experimental house sparrows (~ 8 × 10^5^ mature erythrocytic meronts were inoculated in each recipient). Second, the infected blood from another 3 siskins was collected, mixed and used to infect 8 experimental common chaffinches (~ 5 × 10^5^ mature meronts were inoculated in each recipient). In 2016, blood from one siskin was used to infect 8 experimental common starlings (~ 9.5 × 10^5^ mature meronts were inoculated in each recipient). When parasitaemia developed in the starlings, blood from 3 individuals was mixed and used to infect 8 experimental common crossbills (~ 3 × 10^6^ mature meronts were inoculated into the recipient). Due to unavailability of siskins in 2016, crossbills were used to multiply the strain. Both siskins and crossbills are closely related members of the Fringillidae. Because the parasite strain was the same, it was predicted that the use of different parasite donors would not influence susceptibility of recipient avian hosts or development of phanerozoites.

In all experiments, 100 µl of infected donor blood was mixed with 25 µl sodium citrate and 125 µl 0.9% saline solution per recipient as described by [33]. The mixture was sub-inoculated into the pectoral muscles of the experimental birds. In all, 8 birds of each species were inoculated with *P. homocircumflexum* (lineage pCOLL4). Blood from uninfected common crossbills was inoculated into 8 non-infected birds of each species with the aim to standardize the stress level in experiment and control groups at the start of the experiment. These birds were maintained as control groups at the same conditions as the corresponding species of experimental groups.

The crossbills, house sparrows, chaffinches and starlings, were observed and sampled for 40, 48, 57 and 64 days, respectively. Differences in the period of sampling between different bird species are due to different mortality rates, which were reported within each bird species during this study. Blood from crossbills and starlings was taken for microscopic examination and PCR-based testing every 4 days, and it was obtained from sparrows and chaffinches, starting on 5 DPE, every 3 days during the experiment. Approximately 50 µl of blood was collected in heparinized microcapillaries after puncturing the brachial vein with a sterile needle. Three drops of blood were used to make three blood films, which were air-dried, fixed in absolute methanol, stained with Giemsa and examined microscopically as described by [34]. Approximately 35 µl of the blood was fixed in non-lysis SET buffer (0.05 M Tris, 0.15 M NaCl, 0.5 M EDTA, pH 8.0) for molecular analysis; these samples were stored at − 4 °C in the field and maintained at − 20 °C in the laboratory.

After the birds’ death or euthanasia at the end of the experiment, brain, heart, kidney, liver, lungs, spleen, and a piece of the pectoral muscle of the experimental birds were dissected and fixed in 10% neutral formalin. In the laboratory the collected tissues were embedded in paraffin blocks. Histological sections of 4 µm were prepared, stained with haematoxylin–eosin (H&E) [12] and examined microscopically. Additionally, one thin smear of bone marrow was prepared on a glass slide from each bird. These preparations were processed and examined as the blood films.

### Morphological analysis

An Olympus BX51 light microscope equipped with Olympus DP12 digital camera and imaging software Olympus DP-SOFT was used to examine slides and to prepare illustrations. Each blood slide was examined for 15–20 min at medium magnification (400×), and then at least 100 fields were studied at high magnification (1000×). Intensity of parasitaemia was calculated as a percentage by actual counting of the number of parasites per 1000 erythrocytes or per 10,000 erythrocytes if infections were light [35]. Histological preparations were examined at low magnification (200×) for 10–15 min., followed by examination at medium magnification (400×) for 10–15 min and then at high magnification (1000×) for another 20–30 min.

### Statistical analyses

Statistical analyses were carried out using the ‘R’ package [36]. Normality of data distribution was evaluated using Shapiro–Wilk test. Wilcoxon test was applied for data which were not distributed according to normal distribution in order to evaluate the differences between the means. Fisher’s exact test was used to evaluate if there was a statistically significant difference between mortality in control and experimental group separately in each bird species. Because of different doses of infection in different bird species (Table 1, see also the “Experimental design” section above), mortality rates were not compared between bird species.

### Molecular analysis

Total deoxynucleic acid (DNA) was extracted from collected blood samples using an ammonium-acetate protocol [37] with one modification, 125 µl of fixed blood was used instead of 250 µl. A nested-PCR protocol [38] was applied for the molecular analysis. Primer pair HaemFNI/HaemNR3 was used for the first PCR according condition described by [38]. This primer pair amplifies a partial sequence of the mitochondrial cytochrome b (*cytb*) gene of *Plasmodium*, *Haemoproteus* and *Leucocytozoon* species. Reaction mix for the first PCR consisted of 12.5 µl of Dreamtaq Master Mix (Thermo Fisher Scientific, Lithuania), 8.5 µl of nuclease-free water, 1 µl of each primer and 2 µl of template DNA. For the second PCR, the primer pair HaemF/HaemR2 was used according to the conditions described by [39]. The later primer pair amplifies a 479 bp fragment of *cytb* gene. The reaction mix for the second PCR was as for the first one (only this time using different primers) and instead of genomic DNA, 2 µl of the first PCR product for the second PCR was used. PCR success was evaluated by performing electrophoresis on a 2% agarose gel. 2 µl of the second PCR was used for this evaluation. One negative control (nuclease-free water) and one positive control (a *Plasmodium* sample, which was positive in previous testing) were used to determine possible false amplifications. No case of false amplification was found. Positive PCR products were sequenced from the 5′ end using the HAEMF primer [39]. Dye terminator cycle sequencing (Big Dye) was used. Samples were loaded onto an ABI PRISM TM 3100 capillary sequencing robot (Applied Biosystems, USA). Sequences of parasites were edited and examined using the BioEdit program [40]. The ‘Basic Local Alignment Search Tool’ using the megablast algorithm were applied to identify the *cytb* lineages of detected DNA sequences. Identified sequences were double checked using the ‘Basic Local Alignment Search Tool’ in MalAvi database [41].

### In situ hybridization

Chromogenic in situ hybridization (ISH) was performed on tissue sections that appeared to be non-infected during microscopic examination of H&E preparation. The procedure was carried out according to [26]. In short, paraffin embedded tissue sections of 3 μm thickness were treated in proteinase K (Roche, Basel, Switzerland) 6 μg/ml and Tris-buffered saline solution at 37 °C for about 50 min. Hybridization was carried out overnight at 40 °C with hybridization mixture placed on the histological sections. Concentration of the probe used during the incubation was 100 ng/ml. The probe labelled with digoxigenin at the 3′ end (Eurofins MWG Operon, Ebersberg, Germany) is aimed at 18S ribosomal ribonucleic acid (rRNA) strand and is specific to avian *Plasmodium* spp. [26]. The hybrids were detected by incubating slides with antidigoxigenin-AP Fab fragments (Roche) (1:200) for 1 h at room temperature followed by visualization reaction using the colour substrates 5-bromo-4-chloro-3-indolyl phosphate (BCIP) and 4-nitro blue tetrazolium chloride (NBT) (Roche). Probe specificity has been extensively tested previously [26, 27]. Conducted pilot study [17] showed that *P. homocircumflexum* (lineage pCOLL4) phanerozoites were readily detectable using this method. Tissues from a deceased wild Blackbird *Turdus merula* free of avian malaria parasites were used as a negative control and an irrelevant oligonucleotide probe (designed for *Leishmania* spp.) was applied on the experimental samples to detect any false hybridizations, and tissues of *Plasmodium elongatum* (lineage pERIRUB01) infected canary were used as a positive control.

## Results

**Fig. 1:**
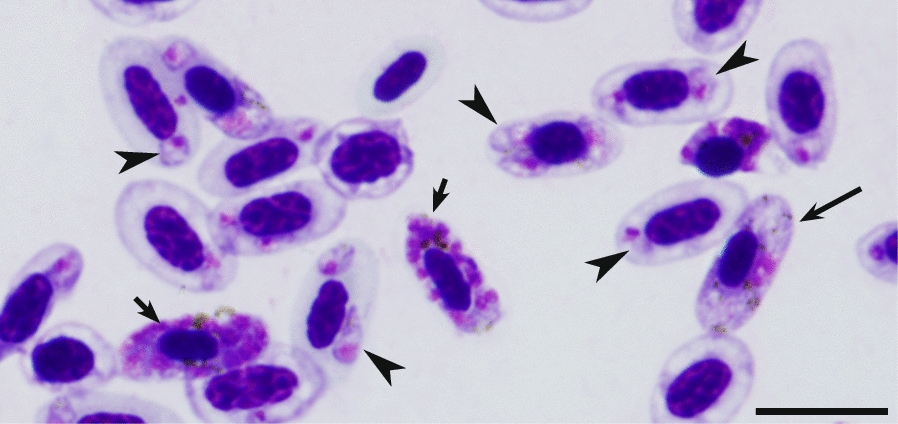
High parasitaemia of *Plasmodium (Giovannolaia) homocircumflexum* (cytochrome b lineage pCOLL4) in an experimentally infected European siskin *Carduelis spinus*. Numerous developing young parasites (simple arrowheads), two mature meronts (short arrows) and one mature macrogametocyte (long arrow) are shown, indicating complete life cycle in avian hosts. Giemsa stained blood film. Scale bar = 10 μm

Both microscopic and PCR-based analyses showed that all birds used in this work were uninfected with haemosporidian parasites prior to experiments. Control birds remained uninfected during this study, indicating that all infection in experimental groups were induced exclusively during experiments. Parasitaemia developed in all experimentally infected birds (Table 1), with mature erythrocytic meronts and gametocytes (Fig. 1) present in all infected individuals. That indicates susceptibility of all exposed bird species. Morphologically indistinguishable blood stages developed in all exposed bird species. Both microscopic examination and PCR-based testing revealed presence of a single infection, pCOLL4 lineage of *P. homocircumflexum* in all experimental birds.

**Fig. 2:**
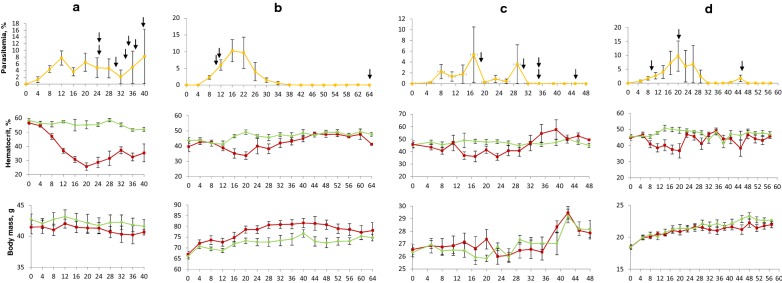
Dynamics of mean parasitaemia of *Plasmodium (Giovannolaia) homocircumflexum* (cytochrome *b* lineage pCOLL4), mean haematocrit value and body mass in experimentally infected (red line) and control (green line) *Loxia curvirostra* (**a**), *Sturnus vulgaris* (**b**), *Passer domesticus* (**c**) and *Fringilla coelebs* (**d**). Arrows indicate the days when individual experimentally infected birds died. Abscissa shows days post exposure. Vertical lines indicate standard error. Note that dose of infection was different in different bird species, and comparison of data between avian hosts should be done with caution

Data on common crossbills’ susceptibility to *P. homocircumflexum* (prepatent period, maximum parasitaemia, mortality rate, development of phanerozoites) are provided in Table 1 and Fig. 2a. Average parasitaemia reached the peak of 7.8% on 12 DPE (Fig. 2a). After the peak, parasitaemia remained and fluctuated in exposed individuals, but average parasitaemia did not reach the peak levels again. Experimental infection had a significant negative effect on the average haematocrit value of common crossbills (p < 0.05) (Fig. 2a). Average haematocrit value of the experimental group decreased more than twofold compared to the control group. The decrease of haematocrit value coincided with high parasitaemia. After the initial decrease, average haematocrit value maintained low and did not reach the initial value. There were no significant average body mass changes in the exposed common crossbills in comparison to controls (p = 0.19) (Fig. 2a). Seven of 8 infected common crossbills died between 25 and 40 DPE (Fig. 2a; Table 1), but all control crossbills survived. Mortality rate in experimental group was significantly higher than in control group (p < 0.001). Phanerozoites were seen in histological sections in all examined organs of the dead common crossbills (Table 1), and they were numerous in brain. Phanerozoites were also observed in the same organs of one crossbill that survived during the experiment, but they were absent in the brain of this individual bird, indicating that the brain pathology is an essential reason of mortality.

Data on common starlings’ susceptibility to *P. homocircumflexum* (prepatent period, maximum parasitaemia, mortality rate, development of phanerozoites) are provided in Table 1 and Fig. 2b. Average parasitaemia reached the peak of 10.2% on 16 DPE (Fig. 2b). After the peak, parasitaemia fluctuated in exposed individuals, but average parasitaemia did not reach the peak level again. Average body mass of the experimental group statistically significantly increased comparing to the control group (p < 0.05) (Fig. 2b). Three of 8 common starlings died during the experiment (8 DPE, 9 DPE and 64 DPE) (Fig. 2b). One common starling died in the control group on the 40 DPE. There was no significant difference in mortality in control and experimental groups (p = 0.57). No phanerozoites were observed in any common starlings—neither dead, nor the survived individuals during histological examination of organs (Table 1), and this result was confirmed by the negative in situ hybridization tests.

**Fig. 3:**
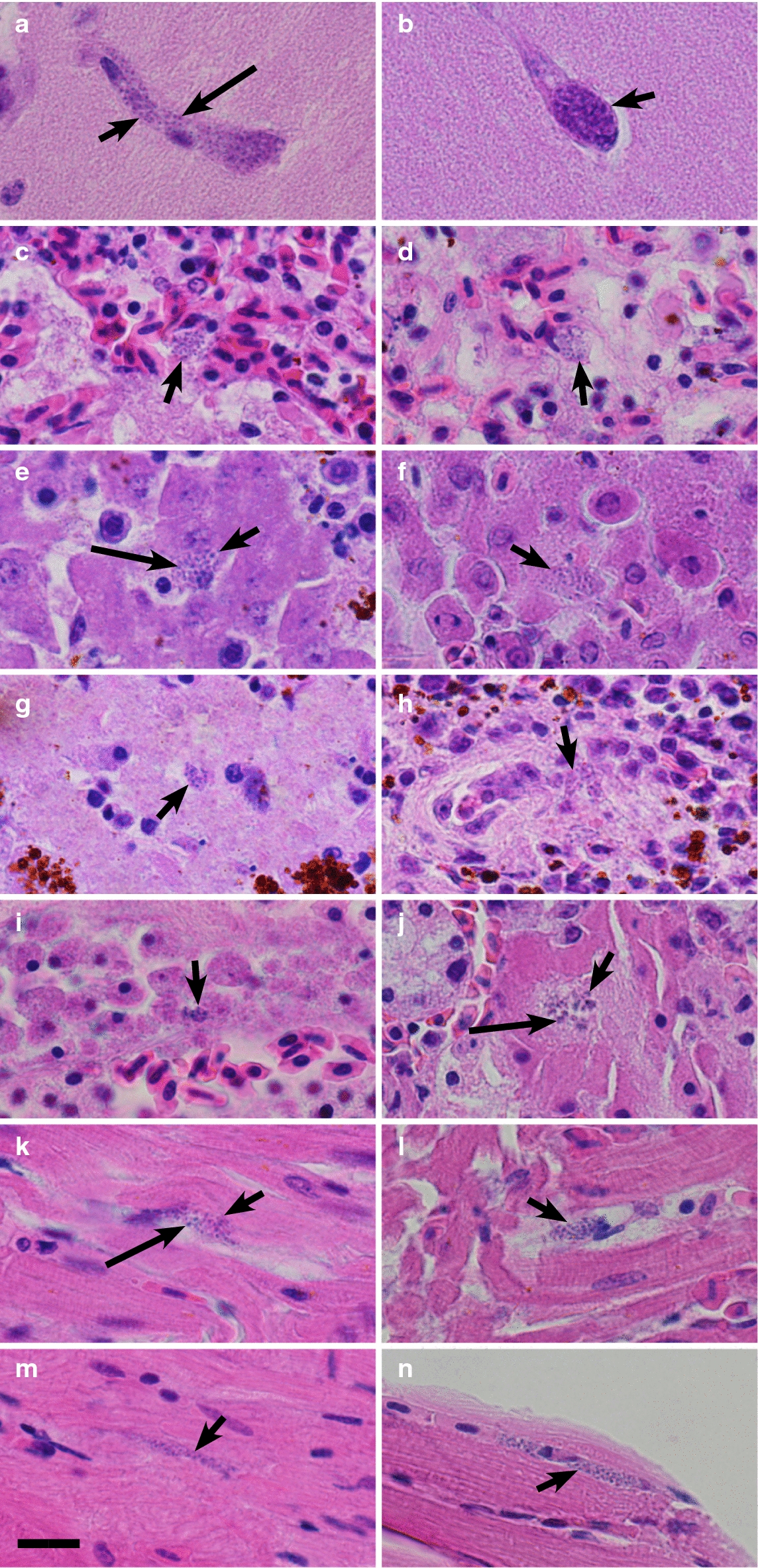
Phanerozoites of *Plasmodium (Giovannolaia) homocircumflexum* (cytochrome *b* lineage pCOLL4) in histological sections of brain (**a**, **b**), lung (**c**, **d**), liver (**e**, **f**), spleen (**g**, **h**), kidney (**i**, **j**), heart (**k**, **l**) and pectoral muscle (**m**, **n**) of experimentally infected *Passer domesticus* (**a**, **c**, **e**, **g**, **i**, **k**, **m**) and *Fringilla coelebs* (**b**, **d**, **f**, **h**, **j**, **l**, **n**). Morphologically similar phanerozoites were reported in all infected experimental birds, but they were absent in starlings. Short arrows: phanerozoites, long arrows: merozoites. Haematoxylin–eosin stained preparations. Scale bar = 20 μm

Data on house sparrows’ susceptibility to *P. homocircumflexum* (prepatent period, maximum parasitaemia, mortality rate, development of phanerozoites) are provided in Table 1 and Fig. 2c. Average parasitaemia reached the peak of 5.7% on 17 DPE (Fig. 2c). After the peak, parasitaemia fluctuated markedly, but average parasitaemia did not reach the peak levels again. Average body mass of exposed house sparrows changed significantly (p = 0.04) (Fig. 2c). Until 20 DPE, the average body mass of the experimental group increased in comparison to controls (p = 0.042). This coincided with the increased parasitaemia. After 20 DPI, the average body mass of the experimental group decreased. It is worth nothing that the decrease in average body mass of the control group was also detected after 20 DPI (Fig. 1). After 34 DPE, the average body mass of both experimental and control group increased sharply. Five of 8 infected sparrows died (Fig. 2c; Table 1). One bird died 18 DPE, others died between 31 and 45 DPE. There was no significant difference in mortality in control and experimental groups (p = 0.31). Phanerozoites were seen in the lungs, liver, spleen and kidney of the experimentally infected house sparrow that died on 18 DPE. In the infected sparrows that died between 31 DPE and 45 DPE, phanerozoites were seen in all examined tissues, including the brain (Fig. 3a, c, e, g, i, k, m). In the house sparrows that survived the experiment, few phanerozoites were located in the lungs, liver and kidney, but were absent in brain. It is important to note that three house sparrows died during light chronic parasitaemia between 36 DPE and 45 DPE, showing that decrease of parasitaemia is not always indication of improved health during avian malaria. On 20 DPE and 43 DPE, two house sparrows from the control group died; malaria parasite were not reported in them.

Data on common chaffinches’ susceptibility to *P. homocircumflexum* (prepatent period, maximum parasitaemia, mortality rate, development of phanerozoites) are provided in Table 1 and Fig. 2d. Average parasitaemia reached the peak of 9.7% on 20 DPE (Fig. 2d). After the peak, parasitaemia fluctuated in exposed individuals, but average parasitaemia did not reach the peak levels again. The decrease in the average haematocrit value were noted on 29 DPE and 44 DPE, which again coincided with increases in average parasitaemia (Fig. 2d). Average body mass of experimental common chaffinches was significantly lower (p = 0.001) than of control group (Fig. 2d). Three infected common chaffinches died on 10 DPE, 21 DPE and 46 DPE. Phanerozoites were observed in all examined tissues of the chaffinches which died on 21 DPE and 46 DPE (Fig. 3b, d, f, h, j, l, n), but no phanerozoites were found in the chaffinch that died on 10 DPE. There was no significant difference in mortality in control and experimental groups (p = 0.57). In chaffinches that survived the experiment, phanerozoites were located in the parenchymal organs (lung, liver, kidney and spleen), and they were absent in brain (Table 1). One control chaffinch died on the 11 DPE; malaria parasites were not reported in this individual.

Phanerozoites observed in the brain were more or less elongate (Fig. 3a, b), and they followed the shape of the brain capillaries. Large phanerozoites filling the entire diameter of the capillaries were common, and they blocked the blood flow (Fig. 3a, b). Merozoites were readily visible in mature phanerozoites (Fig. 3a). Phanerozoites appeared to be roundish in most cases in other organs, such as lung (Fig. 3c, d), liver (Fig. 3e, f), spleen (Fig. 3g) and kidney (Fig. 3i), but phanerozoites of irregular shape were also seen (Fig. 3h, j). Phanerozoites were of oval shape in the heart (Fig. 3k, l), and the ones in the pectoral muscle were mostly slender and elongate (Fig. 3m, n). Inflammatory response to the phanerozoites appeared to be mild or was not seen.

In all experiments, the decrease of the average haematocrit value coincided with increased parasitaemia of *P. homocircumflexum* (Fig. 2). This was a general pattern with minor variations in different species of avian hosts independently of dose of infection. The same lineage of malaria parasite influenced body mass of birds, but markedly differently in different avian host species, ranging from the decrease of body mass in exposed crossbills and chaffinches to the increase in common starlings and house sparrows.

Development of phanerozoites occurred in all exposed avian hosts, except starlings (Table 1). Presence of phanerozoites was associated with brain damage; this stage likely lead to mortality of infected individuals in all experimental groups since the phanerozoites were seen in brain of all dead experimental birds. Starlings were resistant in regard of development of phanerozoites in spite of being inoculated with the second highest dose of infection. In all susceptible experimental birds, the spleen and liver were markedly enlarged, of black colour in comparison to controls.

The dose of infection did not influence the susceptibility of experimental birds, the minimum prepatent period nor the average maximum parasitaemia (Table 1). These parameters were similar in all experimental groups irrespectively on the dose of infection. Development of phanerozoites also was not strictly related to dose of infection because the dose was similar in experimentally exposed house sparrows and common starlings, but phanerozoites developed only in the former species (Table 1). The highest mortality was reported in common crossbills, which were injected with largest dose of parasites.

## Discussion

This study was designed with the aim to describe the pathologies caused by *P. homocircumflexum* (lineage pCOLL4) in different species of avian hosts. Due to complicated methodology of strain multiplication and calculation of the number of mature meronts in inoculated blood during different experiments, it was impossible to standardize and calculate the dose of infection in each experiment precisely. This shortcoming prevents detailed comparison quantitative data between different avian hosts. However, the following key findings are innovative, are not related to dose of infection and should be discussed. First, *P. homocircumflexum* (lineage pCOLL4) developed high parasitaemia in all exposed wild passerine birds, indicating broad specificity and potentially big invasive ability in regard to vertebrate host range. Second, this parasite is virulent, with high maximum parasitaemia reported in all exposed birds. Third, general pattern of parasitaemia dynamics and haematocrit value changes were similar in all exposed birds. Fourth, phanerozoites developed in all bird species, except starlings, resulting in different mortality rates. It is also interesting to note that this infection influenced body mass of birds, but markedly differently in different host species; however, the reported differences might be related to different dose of infection and should be treated with caution.

This study shows that *P. homocircumflexum* (lineage pCOLL4) is able to infect and develop parasitaemia in distantly related passeriform bird species belonging to different families. Both the susceptibility and minimum prepatent period as well as maximum average parasitaemia were similar in different bird species without relation to the inoculated dose of mature erythrocytic meronts. All individual birds belonging to the Fringillidae, Passeridae and Sturnidae families were susceptible to this infection, indicating that *P. homocircumflexum* is truly a generalist parasite. This observation is in accordance with PCR-based records of this parasite lineage in wild birds. In all, *P. homocircumflexum* was reported in birds belonging to 14 species ([8, 17, 31, 42], present study). However, only in 7 bird species (*Lanius collurio*, *Serinus canaria*, *Carduleis spinus*, *Loxia curvirostra*, *Sturnus vulgaris*, *Passer domesticus* and *Fringilla coelebs*) mature gametocytes were observed (Fig. 1), indicating completion of life cycle in these avian hosts and the potential ability of the parasite to infect vectors ([8, 17], present study). In other published reports, the lineage pCOLL4 or synonymous lineages were detected only by PCR-based analysis [31, 42], and it was unclear if this infection completes or aborts development in the reported PCR-positive individuals. Abortive haemosporidian infections seem to be common in wildlife but are dead ends of haemosporidian parasite transmission [43]. The pathogen’s ability to infect the broad range of vertebrate hosts and produce infective stages (gametocytes) is an important point to consider in regard to epidemiology of this infection. Vectors species of *P. homocircumflexum* (pCOLL4) remain unknown. Running hypothesis is that migrating European birds are naturally infected in Africa, but transmission might be interrupted due to lack of susceptible mosquito species in Europe [8]. Because common European birds are readily susceptible, get sick and often die (Table 1), further research is essential for better understanding true infection prevalence in wildlife populations and mechanisms preventing transmission of this parasite at breeding grounds of European birds. It is worth noting that experimentally infected birds in the present study are sedentary or short-distance migrants.

Further studies in these bird local populations as well as in the blood of the juveniles of long-distance migrants (for example, the red-backed shrike from which the strain was originally isolated) in Europe are needed to prove or reject the running hypothesis. It is possible that the available data about low prevalence of *P. homocircumflexum* (pCOLL4) might indicate high mortality of susceptible birds in the wild. The lack of suitable vector might also be an important limiting factor at present. However, it is difficult to predict how the epidemiological situation would develop due to climate change and spread of new mosquito species in Europe [44, 45]. That calls for epidemiological research of *P. homocircumflexum* (pCOLL4).

This study supplements the results of [20] experiments, in which 5 species of passeriform birds (common crossbills, common chaffinches (*Fringilla coelebs*), common starlings, house sparrows and Eurasian siskins) were exposed to *Plasmodium relictum* (lineage pSGS1) infection, but showed different dynamics of parasitaemia and parasitaemia related haematocrit and body mass changes. Interestingly, the susceptibility of these bird species to *P. relictum* was markedly different [20], but it was the same during infection of *P. homocircumflexum* in this study, in which all individuals of all bird species developed parasitaemia. Additionally, the common starlings were more resistant to *P. relictum* in comparison to other bird species in both experiments. However, in this study, all common starlings were susceptible to *P. homocircumflexum* infection and developed parasitaemia, but this bird species was completely resistant to *P. relictum* with no parasite reported in blood [20].

In this study, the partial resistance of starlings to *P. homocircumflexum* was manifested not in parasitaemia, but in absence of phanerozoites in all exposed birds (Table 1). In other words, the available experimental observations indicate that the development of the same pathogen might be different in different species of avian hosts not only on the parasitaemia stage, but also on the exo-erythrocytic stage. The mechanisms responsible for the observed differences in parasite development in different species of birds remain unclear, and they might be due to the species-related innate resistance, which varies between different bird species [46–48]. However, immunity issues remain insufficiently investigated, and belong to the weakest understood points of avian malaria infections [12]. It is unclear how the avian immune system combats infections and how various biological factors (stress, co-infections, previous diseases, nutrition) influence immunity [49, 50]. Further experimental studies are needed for better understanding how immunological factors affect the success of parasitic infections. The host-parasite models developed in this study provide theoretical backgrounds and experimental opportunities in sampling materials for addressing comparative immunological research.

Avian malaria is a disease that causes blood pathology due to direct destruction of erythrocytes [11, 12] or damage of stem cells in bone marrow, leading to interruption of erythropoiesis [8, 11, 51]. During this study, parasites were not reported in stem cells of bone marrow, but high parasitaemia developed in the majority of birds (Table 1), indicating the anemia due to direct destruction of red blood cells by developing parasites and their removal in spleen and liver, which were enlarged, of black color and overfilled by infected erythrocytes and pigment granules in all dissected sick birds. The haematocrit values of the exposed birds decreased significantly during high parasitaemia in all tested bird species. This finding agrees with reports of former studies with different *Plasmodium* species in different species of birds [20, 52–54]. Interestingly, in the case of house sparrows, the decrease of haematocrit value was overcompensated and even exceeded that of the control group of the same species in the end of experiment (Fig. 2c). The greatest effect on haematocrit value was reported in infected common crossbills (Fig. 2a), and this suggests not only direct destruction of erythrocytes by parasites, but also active removal of infected erythrocytes by cells of the reticuloendothelial system in the spleen and liver. In the cases of common starlings and common chaffinches, the changes of haematocrit value were strictly positively correlated with the increase of parasitaemia, and then this parameter returned to normal levels when parasitaemia decreased. These results agree with results from previous studies measuring the haematocrit value in experimentally infected birds [20, 55].

Unexpectedly, infected common starlings and house sparrows showed a significant increase in body mass (Fig. 2b, c). This could be related to the availability of food: all birds were fed *ad libitum* and sick birds might eat more than controls. Our preliminary visual observations on control and infected birds support this hypothesis however, the food consumption was not measured during the study. It is probable that the same result hardly would be achieved in nature where the food supply is limited due to competition and the threat of predators. It is interesting to note that the infected common starlings were particularly active to the offered food during this study, and they were observed starting to eat even while the feeder’s hand was still holding the feeder in the cage. In other words, they were not afraid of people during feeding. Similar behaviour observation has been reported in previous study [20] where the exposed Eurasian siskins were not scared by people entering the room and continued consuming food. It might be that some species of birds increase food consumption as a compensatory mechanism during loss of energy during malaria. However, it seems this is not the case in all bird species because chaffinches along with crossbills were not seen to increase the food consumption and their body mass did not increase in comparison to controls (Fig. 2a, d), which might be an indication of a possible existence of species-related mechanisms responsible for food consumption [56]. This study shows that increased body mass is not always an indication of good health during avian malaria.

This is the first study, which reports clear differences in exo-erythrocytic development of the same parasite lineage in different avian hosts. In other words, it was documented that not only susceptibility and parasitaemia dynamics, but also exo-erythrocytic development of the same pathogen might be different in different species of avian hosts. Phanerozoites developed in 3 species of birds infected with different doses of parasite, but they did not appear in common starlings (Table 1). It seems that the phanerozoites of *P. homocircumflexum* (pCOLL4) start to develop around 20 DPE in experimentally infected birds because phanerozoites were not seen in the chaffinch that died on 10 DPE but were observed in all tissues of the chaffinch that died 21 DPE. There might be individual variation in timing of phanerozoite formation, but phanerozoites likely appeared in the tissues between the 10 DPE and 21 DPE. In other words, some period of parasite adaptation to the avian host is needed before merozoites acquire ability to inhabit reticuloendothelial cells in organs. In dead house sparrows, phanerozoites were seen in lungs, liver, spleen and kidney on the 18 DPE, and they were present in all organs, including the brain 31 DPE. Common crossbills started dying 25 DPE during the experiment, and phanerozoites were seen in all of the examined tissues. These results second and expand on the results by [8, 17] who reported phanerozoites of *P. homocircumflexum* in all examined organs of birds which died 19 DPE and 38 DPE. Interestingly, phanerozoites were observed in the brain of all birds that died starting from 21 DPE until the end of the experiment, except for the starling, in which no phanerozoites developed. Phanerozoites in the brain were not observed in any of the birds that survived the experiment. This suggests that phanerozoites in the brain most likely cause the death in all *P. homocircumflexum* malaria cases, as was determined by prior works in some other malaria parasites [12].

Several birds from control groups died during this study, and this partly complicates the understanding of the experimental results. Two of the control house sparrows, one control common chaffinch and one control common starling died during this long-lasting experiment (Table 1), indicating that maintaining of wild birds in captivity and experimental manipulations are stressful for them. This raises the question whether the all deaths observed in the experimental groups were truly related to the effects of malaria or were, they—at least in part—caused by other unknown factors. The experiment with common crossbills was most successful, and it could help answering this question. Mainly, 7 of 8 infected common crossbills died during the experiment (p < 0.001) and phanerozoites were seen in every examined organ, including the brain. One of 8 crossbills with parasitaemia of approximately 8% survived the experiment; it was euthanized and phanerozoites were seen in all examined organs except for the brain. Also, none of the control group crossbills died. This allows the assumption, that most likely phanerozoites in the brain, along with damage to parenchymal organs were the cause of death of the experimental birds. It seems probable that even though several control group birds died in the cases of house sparrows, common starlings and common chaffinches (behaviour of these birds was particularly stressful in captivity), it is likely that phanerozoites of *P. homonucleophilum* developing in the brain are essential reason of mortality during this study.

It is important to note that in the case of common starlings, results of virulence differed from those obtained during our former pilot study [17] during which only one juvenile common starling was exposed to *P. homocircumflexum* infection. Development of the parasitaemia followed a similar pattern in both experiments. Mainly, after similar prepatent period, the parasitaemia developed to reach a peak and then decreased, eventually turning into a chronic stage. On 36 DPE of the pilot study the common starling suddenly died. Phanerozoites were located in the examined organs (brain, heart, liver, lung, kidney, spleen and a piece of the pectoral muscle) and positive ISH result confirmed that these exo-erythrocytic meronts were correctly identified as *Plasmodium* phanerozoites. That was not the case in this study. Two starlings died very early in the experiment (8 DPE and 9 DPE). Histological and ISH examination did not reveal any developed exo-erythrocytic stages. The low parasitaemia and absence of parasites in tissues suggest that these two individuals died of factors other than malaria. Even though parasitaemia developed in surviving birds in this study, as was also indicated by the pilot study—it reached a peak and turned into chronic stage, but exposed birds survived longer than expected according to the pilot study. Only one common starling infected with *P. homocircumflexum* died on 64 DPE. After the end of the experiment, all infected starlings were examined histologically. Contrary to the pilot study, no phanerozoites were detected in neither the surviving starlings, nor the one that died during the experiment. The negative histological results were confirmed by ISH tests performed in all examined organs of all experimental starlings. This raises a question for the reason of this difference is in these two experiments. Previous studies have suggested that common starlings can resist *P. relictum* (lineage pSGS1) infection [12, 20]. Since the starling in the pilot study was caught from the wild, it is impossible to know if it had not contracted some immune system suppressing disease prior to the experiment in the pilot study. This result also suggests that there might be marked individual variation in susceptibility of birds to support exo-erythrocytic development, which initiation mechanism remains unclear. The intriguing question remains, why common starlings are able to resist malaria *P. relictum* and *P. homocircumflexum* infections. This avian host and the parasites might be used as model organisms to study molecular mechanisms of innate resistance during avian malaria.

## Conclusion

Contrary to the common belief, bird malaria parasites cause not only severe blood pathology, but also inhabit and damage various internal organs including brain, heart, liver, lungs, kidneys and spleen. Knowledge about the development of tissue stages remain insufficient during avian malaria, especially in wildlife. This study shows that wild birds belonging to three families (house sparrows, chaffinches, common crossbills and common starlings) are highly susceptible to *P. homocircumflexum* infection, which is broad generalist malaria parasite. Mortality due to malaria was reported in three of the four exposed bird species, and the brain damage due to cerebral ischemia caused by phanerozoites was associated with mortality in the majority of exposed birds. This finding contributes to better understanding the pathology during avian malaria infections and indicate possible directions for development of treatment, which must address not only blood stages, but also tissue stages of the parasite, which damage organs all over the body of birds.

## Data Availability

The datasets used and/or analysed during the current study are available from the corresponding author on reasonable request.

## Abbreviations

BCIP: 5-bromo-4-chloro-3-indolyl phosphate
*cytb*: mitochondrial cytochrome *b*
DNA: deoxynucleic acid
DPE: days post exposure
H&E: haematoxylin–eosin
ISH: chromogenic in situ hybridization
NBT: 4-nitro blue tetrazolium chloride
PCR: polymerase chain reaction
rRNA: ribosomal ribonucleic acid
